# Data on the natural ventilation performance of windcatcher with anti-short-circuit device (ASCD)

**DOI:** 10.1016/j.dib.2016.08.042

**Published:** 2016-08-28

**Authors:** Payam Nejat, John Kaiser Calautit, Muhd Zaimi Abd. Majid, Ben Richard Hughes, Fatemeh Jomehzadeh

**Affiliations:** a Faculty of Civil Engineering, Universiti Teknologi Malaysia, UTM, Skudai, Johor, Malaysia; b Department of Mechanical Engineering, University of Sheffield, Sheffield, UK; c Construction Research Center, Institute of Smart Infrastructure and Innovative Construction, Universiti Teknologi Malaysia, Johor, Malaysia; d Advanced Built and Environment Research (ABER) center, Tehran, Iran

## Abstract

This article presents the datasets which were the results of the study explained in the research paper ‘Anti-short-circuit device: a new solution for short-circuiting in windcatcher and improvement of natural ventilation performance’ (P. Nejat, J.K. Calautit, M.Z. Abd. Majid, B.R. Hughes, F. Jomehzadeh, 2016) [1] which introduces a new technique to reduce or prevent short-circuiting in a two-sided windcatcher and also lowers the indoor CO_2_ concentration and improve the ventilation distribution. Here, we provide details of the numerical modeling set-up and data collection method to facilitate reproducibility. The datasets includes indoor airflow, ventilation rates and CO_2_ concentration data at several points in the flow field. The CAD geometry of the windcatcher models are also included.

**Specifications Table**

**Table:** 

Subject area	*Environmental Science*
More specific subject area	*Air quality, Building, Natural ventilation, Simulation*
Type of data	*Graphs, tables, geometry*
How data was acquired	*ANSYS FLUENT*
Data format	*Raw, analysed*
Experimental factors	*The computational domain was sized based on best practice guideline COST 732. Lateral and vertical extension of the domain was 5H (5 times the height of the building). For the extension of the domain in the direction of flow, 5H used for inlet. For the outlet, the boundary was positioned 15H behind the building. Atmospheric boundary layer (ABL) approach flow profile were imposed on the inlet of the domain. For carbon dioxide concentration simulation, simplified exhalation model*[8]*was used.*
Experimental features	*The CFD code ANSYS FLUENT was used to solve the governing equations (continuity, momentum and energy) in the computational domain using the finite volume method. The second-order upwind scheme was used for numerical discretization along with SIMPLE algorithm. The standard k-*ϵ*turbulence model was used*.
Data source location	*UK*
Data accessibility	*Data of current article*

**Value of the data**

• The computational data can be used to verify modeling predictions of the ventilation rates, airflow and CO_2_ concentration distributions in buildings with a windcatcher.
• The data can be used for comparing with the results of other׳s simulation model by providing a benchmark.
• It can be used for CFD user training and improvement of the accuracy of numerical simulations of windcatchers.
• It can be employed to assess various design optimization of the windcatcher and anti-short-circuit device.

## 1 Data

This article presents the supply and exhaust rates of a windcatcher with “anti-short-circuit device (ASCD)” and also the indoor airflow and carbon dioxide concentration data at several points in the test room incorporating [1]. Four two-sided windcatcher designs were used: benchmark windcatcher, windcatcher with 30° ASCD, windcatcher with 60° ASCD and windcatcher with 90° ASCD. To save time and effort, the geometry files are also attached in Supplementary 2.

The supplementary file includes the following data:

**Air short-circuit data** (Supplementary Table 1) – This dataset provides values of the airflow velocity (Y- direction) obtained from the plotted points between the supply and exhaust channels below the windcatcher for various ASCD angles.

**Ventilation rates data** (Supplementary Table 2) – This dataset provides values of airflow velocity obtained from the plotted points inside the supply channel of the windcatcher for various ASCD angles.

**Airflow distribution data** (Supplementary Table 3) – This dataset provides values of airflow velocity obtained from the plotted points inside the test room for various ASCD angles.

**Carbon dioxide concentration data** (Supplementary Table 4) – This dataset provides values of CO_2_ concentration obtained from the plotted points inside the test room for various ASCD angles.

**CAD Geometry files** (Supplementary 2) **–** The dataset provides CAD geometry files of four configurations of the two-sided windcatcher: standard windcatcher, windcatcher with 30° ASCD, windcatcher with 60° ASCD and windcatcher with 90° ASCD. The windcatcher is integrated to a test room which is surrounded by macro (outdoor) domain.

## 2 Experimental design, materials and methods

**Fig. 1:**
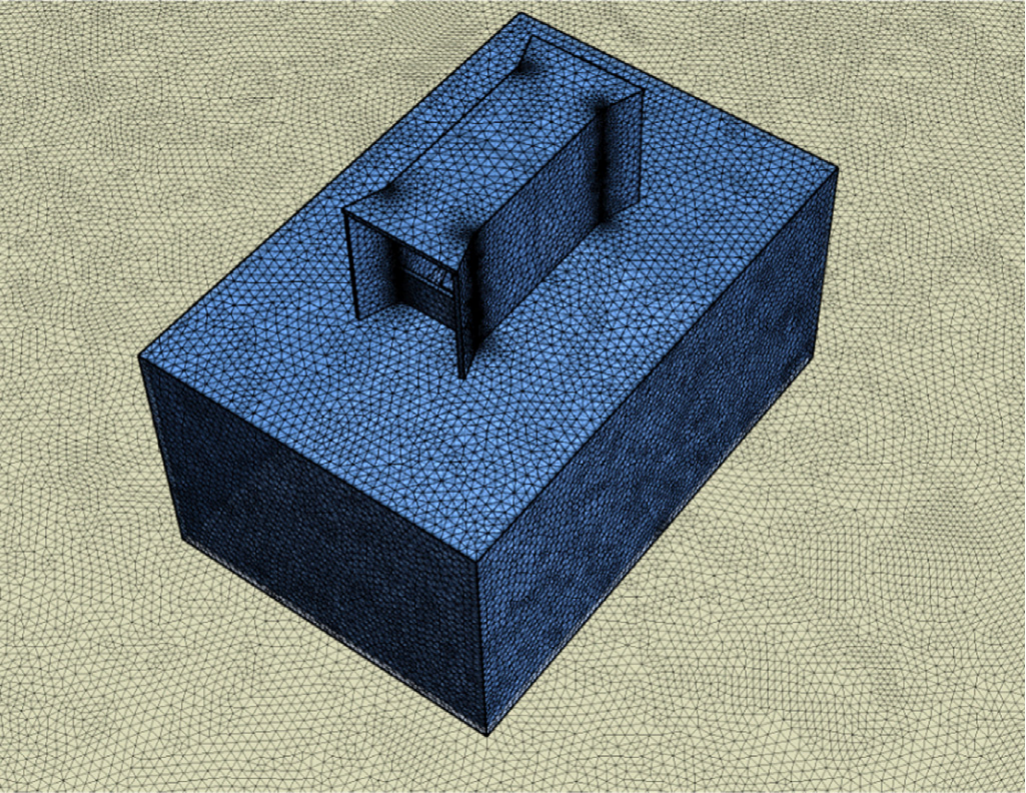
Surface mesh of the windcatcher and room model.

**Fig. 2:**
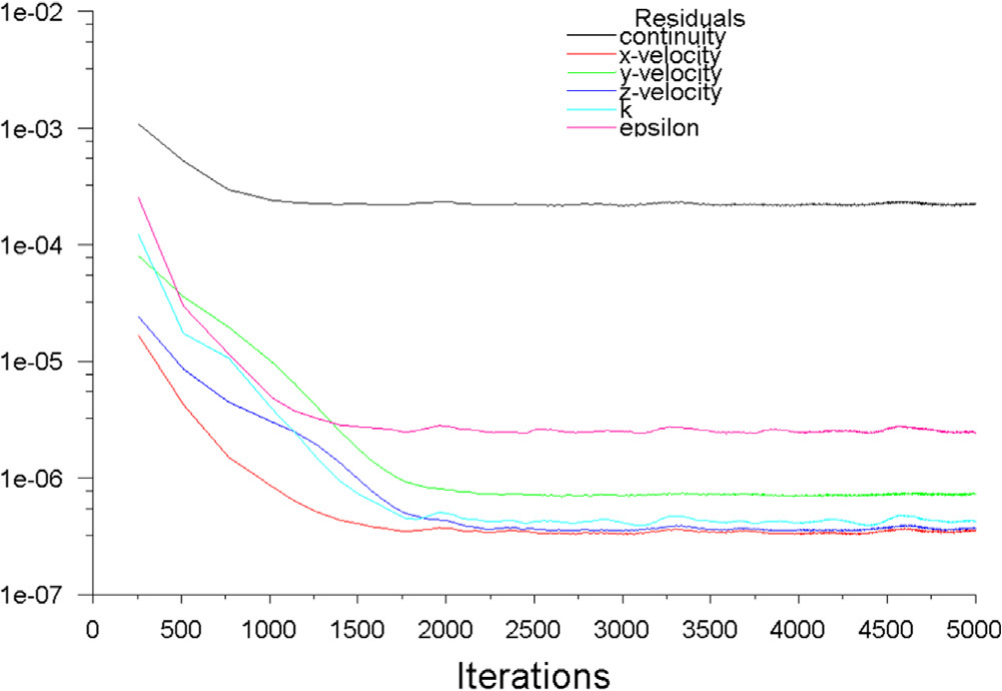
Convergence monitoring of continuity, velocity, *k* and epsilon.

**Fig. 3:**
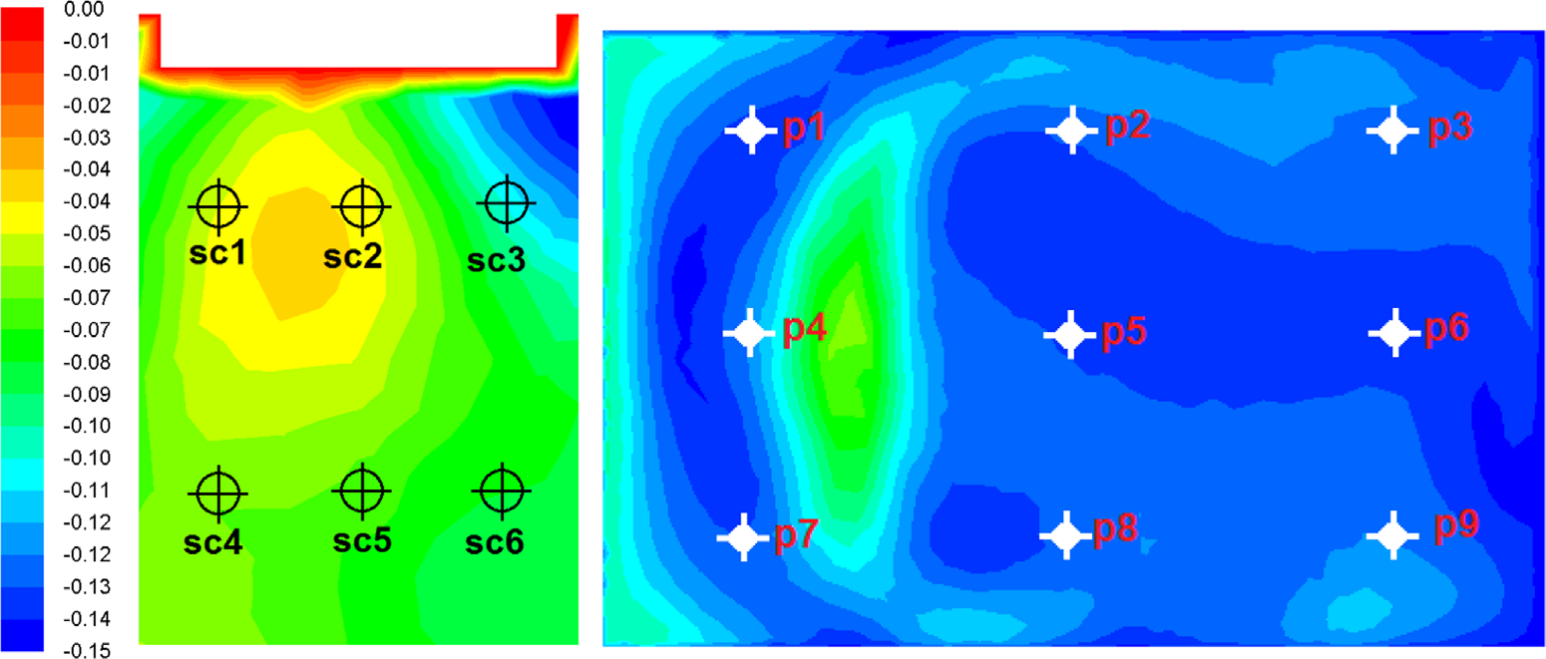
Measurement points for the (a) airflow short-circuiting, (b) CO_2_ concentration and airflow distribution.

A discretized Computational Fluid Dynamics (CFD) code ANSYS FLUENT was used to solve the governing equations (continuity, momentum and energy) in the computational domain using the finite volume method. The second-order upwind scheme was used for numerical discretization along with SIMPLE algorithm. The standard k-ϵturbulence model was used, which is a well-established method in simulations of natural ventilation such as windcatchers [2], [3], [4], [5], [6], [7]. The computational domain consisted of a single fluid zone combining the indoor and outdoor. For meshing the domain, unstructured mesh techniques was used. The domain was sized based on best practice guideline COST 732. Lateral and vertical extension of the domain was 5H (5 times the height of the building). For the extension of the domain in the direction of flow, the inlet boundary was positioned 5H windward of the building. For the outlet, the boundary was positioned 15H behind the building. Atmospheric boundary layer (ABL) approach flow profile which obeyed a power-law with *α*=0.25 were imposed on the inlet of the domain. Pressure outlet was set as atmospheric. The top and side walls were set as symmetry. For carbon dioxide concentration simulation, a simplified exhalation model [8] was used. The total number of grid was approximately 8 million elements (Fig. 1). For the calculations, the computer used 12 CPUs and 16GB of memory. The simulations were completed when additional iterations did not show further variations in the monitored data (Fig. 2). Data were obtained by plotting points inside the windcatcher for the ventilation rates and test room for airflow short-circuiting (sc1–sc6, see Fig. 3a), CO_2_ concentration and airflow distribution (p1–p9, see Fig. 3b).
